# Analysis of the noncoding RNA regulatory networks of H37Rv- and H37Rv△1759c-infected macrophages

**DOI:** 10.3389/fmicb.2023.1106643

**Published:** 2023-03-08

**Authors:** Wenqi Dong, Gaoyan Wang, Yajuan Bai, Yuxin Li, Xinyu Huo, Jing Zhao, Wenjia Lu, Hao Lu, Chenchen Wang, Xiangru Wang, Huanchun Chen, Chen Tan

**Affiliations:** ^1^State Key Laboratory of Agricultural Microbiology, College of Veterinary Medicine, Huazhong Agricultural University, Wuhan, Hubei, China; ^2^Hubei Hongshan Laboratory, Wuhan, Hubei, China; ^3^WuHan Animal Disease Control Center, Wuhan, Hubei, China

**Keywords:** Mycobacterium tuberculosis, noncoding RNA, ceRNA, Rv1759c, chemokines

## Abstract

Noncoding RNAs regulate the process of Mycobacterium tuberculosis (M. tb) infecting the host, but there is no simultaneous transcriptional information of long non-coding RNAs (lncRNAs) and circular RNAs (circRNAs) and the global regulatory networks of non-coding RNA. Rv1759c, a virulence factor, is a member of protein family containing the proline-glutamic acid (PE) in M. tb, which can increase M. tb survival. To reveal the noncoding RNA regulatory networks and the effect of Rv1759c on non-coding RNA expression during M. tb infection, we collected samples of H37Rv- and H37Rv△1759c-infected macrophages and explored the full transcriptome expression profile. We found 356 mRNAs, 433 lncRNAs, 168 circRNAs, and 12 miRNAs differentially expressed during H37Rv infection, 356 mRNAs, 433 lncRNAs, 168 circRNAs, and 12 miRNAs differentially expressed during H37Rv△1759c infection. We constructed lncRNA/circRNA-miRNA-mRNA regulatory networks during H37Rv and H37Rv△1759c infection. We demonstrated the role of one of the hubs of the networks, hsa-miR-181b-3p, for H37Rv survival in macrophages. We discovered that the expression changes of 68 mRNAs, 92 lncRNAs, 26 circRNAs, and 3 miRNAs were only related to the deletion of Rv1759c by comparing the transcription profiles of H37Rv and H37Rv△1759c. Here, our study comprehensively characterizes the transcriptional profiles in THP1-derived-macrophages infected with H37Rv and H37Rv△1759c, which provides support and new directions for in-depth exploration of noncoding RNA and PE/PPE family functions during the infection process.

## 1. Introduction

Tuberculosis (TB) is an important infectious disease threatening the world (Dean et al., 2022). Mycobacterium tuberculosis (M. tb) is the pathogen that causes tuberculosis, and it can survive and replicate in macrophages by evading host immunity with the help of virulence factors (Ge et al., 2021). The genes encoding proteins containing the proline-glutamate (PE) or proline-proline-glutamate (PPE) are the virulence genes that account for 10% of the H37Rv genome. These proteins were classified into PE_PGRS and PE subfamilies based on their characteristic conserved N-terminal PE motif and C-terminal diversity (polymorphic GC-rich sequences or no significant features; Cole et al., 1998). As virulence factors, PE_PGRS family proteins are closely related to the formation of granulomas, immune response of the host, and the pathogenesis of TB (Fishbein et al., 2015). Based on this fact, PE_PGRS family proteins are candidates for vaccine design and show effective protection (Ramakrishnan et al., 2000). Rv1759c belongs to the PE_PGRS family, which located on the cell wall of M. tb with fibronectin-binding activity can prevent TB reactivation in a mouse model as a component of the subunit vaccine (Espitia et al., 1999; Campuzano et al., 2007). Our previous study found that deletion of Rv1759c reduced M. tb intracellular survival and affected miR-25 expression through NF-κB inhibitor ζ (NFKBIZ; Dong et al., 2022). However, the effect of Rv1759c on other host noncoding RNAs and mRNAs during M. tb infection remains unclear.

Noncoding RNAs (ncRNAs) that do not code for proteins include microRNAs (miRNAs) of approximately 22 nucleotides in length, long noncoding RNAs (lncRNAs) of more than 200 nucleotides in length that are transcribed by RNA polymerase II (Pol II), and single-strand covalently closed circular RNA (circRNA) without 5′ cap and 3′ poly-A tail (Bartel, 2004; Xie et al., 2005; Barrett and Salzman, 2016; Ransohoff et al., 2018). Competing endogenous RNAs (ceRNAs), including lncRNAs and circRNAs, can complete the regulation of downstream target genes by competitively binding miRNAs (Tay et al., 2014). Previous studies showed that ncRNAs were involved in important signaling pathways such as autophagy, apoptosis, immunity, and ROS generation during M. tb infection. miR-27a was found to be upregulated during M. tb infection by targeting CACNA2D3 to inhibit autophagosome formation and increase M. tb survival (Liu et al., 2018). It was demonstrated that miR-342 can control susceptibility to M. tb infection in mice and humans by increasing the production of inflammatory cytokines and the cell death pathway through the miR-342/SOCS6 axis (Fu et al., 2021). Studies showed that the expression level of lncRNA-CD244 in the CD244 + CD8+ T cell subsets of active TB (ATB) patients is higher than that of healthy people, and silencing lncRNA-CD244 can increase the production of IFN-γ and TNF-α (Wang Y. et al., 2015). Downregulated lincRNA-EPS in monocytes from ATB patients can inhibit apoptosis of BCG-infected macrophages and promote autophagy (Ke et al., 2020). The literature proved that the upregulated lncRNA MLAT during BCG infection can bind to miR-665 as a ceRNA to attenuate the expression of the target gene ULK1, promote autophagy, and limit the survival of BCG cells (Jiang et al., 2021). CircTRAPPC6B was downregulated during M. tb infection and promoted M. tb growth by inhibiting autophagosome formation through binding miR-874-3p and repressing target gene ATG16L1 (Luo et al., 2021). The increased expression of circAGFG1 in alveolar lavage fluid of ATB patients can inhibit the apoptosis of M. tb-infected macrophages and promote autophagy through the circAGFG1/miR-1257/Notch2 axis (Shi et al., 2020). NcRNAs also have the potential to serve as diagnostic markers, therapeutic targets or therapeutic evaluation indicators for TB (Davuluri and Chauhan, 2022; Hemati et al., 2022; Liang et al., 2022; Wang L. et al., 2022; Wang Q. et al., 2022; Zhang et al., 2022). Studies found that the upregulated hsa_circ_0001953, hsa_circ_0009024, hsa_circ_051239, hsa_circ_029965 and hsa_circ_404022 and the downregulated hsa_circ_0001380 and hsa_circRNA_103571 in the serum and the downregulated NONHSAT101518.2, NONHSAT067134.2, NONHSAT148822.1, and NONHSAT078957.2 in serum exosomes could be used as potential biomarkers for patients with ATB disease (Huang et al., 2018; Yi et al., 2018; Luo et al., 2020; Fang et al., 2021). At present, the data of the transcriptome of lncRNAs and circRNAs are relatively limited. There are no related studies on the simultaneous expression of lncRNA and circRNA. Most of the literatures only analyze the differential expression of lncRNAs or circRNAs in clinical TB samples or laboratory M. tb-infected samples, or explore the regulatory mechanism based on a single differentially expressed lncRNA or circRNA (Roy et al., 2018; Yi et al., 2018; Huang et al., 2020; Fang et al., 2021; Kaushik et al., 2021). And the construction non-coding RNA regulatory networks based on website prediction cannot fully reflect the real regulatory mechanism in the process of M. tb infection.

Macrophages are the primary innate immune cells that clear intracellular pathogens, including M. tb (Weiss and Schaible, 2015). The THP-1 cell line obtained from the peripheral blood of patient with acute monocytic leukemia is widely used in studies related to M. tb (Tsuchiya et al., 1980). THP-1-induced and differentiated macrophages can be used as an infection model for screening anti-tuberculosis drugs, examining the activity of specific pathways, and characterizing the gene expression profile changes through proteomic and transcriptomic during M. tb infection (Fontán et al., 2008; Li et al., 2017; Shah et al., 2022). We collected H37Rv- and H37Rv△1759c-infected THP1-derived-macrophages samples for whole transcriptome sequencing and verified the differentially expressed genes. Subsequently, we screened the lncRNAs, circRNAs, and mRNAs predicted to interact with miRNAs from the differentially expressed genes, and constructed the non-coding RNA regulatory networks during H37Rv and H37Rv△1759c infection, respectively. And we explored the influence of Rv1759c on host mRNAs and non-coding RNAs expression.

## 2. Materials and methods

### 2.1. Bacterial strains

We used pYUB004S and phAE159 to prepare phages containing the upstream and downstream homologous arms of Rv1759c gene and infected H37Rv. After obtaining the gene-deleted strain, the dissociation enzyme Tnp R were introduced and generate H37Rv△1759c without sacB-hyg tag (Bardarov et al., 2002; Jain et al., 2014).

H37Rv and H37Rv△1759c were recovered on solid Middle Brook 7H11 medium (Becton Dickinson, 212203) supplemented with 10% (oleic-albumin-dextrose-catalase) OADC, and 0.5% glycerol. Individual colonies of H37Rv and H37Rv△1759c were picked and cultured in Middlebrook 7H9 medium (Becton Dickinson, 271310) supplemented with 10% OADC, 0.5% glycerol, and 0.05% Tween 80 (Sigma Aldrich, P8074).

### 2.2. Cell culture and bacterial infection

THP-1 cells were cultured in RPMI-1640 + 10% FBS medium, supplemented with phorbol myristate (PMA; 100 ng/mL) for 48 h during differentiation induction. Adherent THP-1 cells were serum-starved for 8 h before infection and infected with H37Rv or H37Rv△1759c at an MOI of 1 for 4 h, and then washed three times with PBS to remove bacteria. There were three replicates of H37Rv, H37Rv△1759c infection, and uninfected cell samples. THP-1 cells were cultured for another 20 h and lysed with TRIzol reagent (Life Technologies, 15596026; Dong et al., 2022). The three comparison groups are Control vs. WT, Control vs. △1759c, and WT vs. △1759c. THP-1 cells were infected with H37Rv at an MOI of 5 for 4 h after induction of differentiation, and samples were collected 20 h post infection to detect the expression of hsa-miR-181b-3p.

### 2.3. Intracellular survival of bacteria

To knock down hsa-miR-181b-3p, we constructed pLVX-sh-hsa-miR-181b-3p plasmid containing the shRNA sequence hsa-miR-181b-3p and pLVX-sh-NC containing the nonsense shRNA sequence. HEK293T cells were transfected with psPAX2, pMD2.G and pLVX-sh-hsa-miR-181b-3p/pLVX-sh-NC to produce lentivirus. sh-hsa-miR-181b-3p and sh-NC THP-1 cell lines were obtained after lentivirus infection and puromycin (InvivoGen, ant-pr-1) screening. The same amount of sh-hsa-miR-181b-3p and sh-NC THP-1 cells were differentiated and infected with H37Rv at an MOI of 5 for 4 h. Cells were washed with PBS and incubated to 12, 24, and 48 h post infection. THP-1 cells were washed with PBS and lysed with 0.025% Triton X-100. The intracellular bacteria were diluted in PBS and plated for counting (Dong et al., 2022).

### 2.4. Transcriptome assembly and differential gene analysis

Total RNA was extracted using RNeasy Mini Kit (Qiagen, 217004) and broken into approximately 300 bp fragments. After obtaining double-stranded DNA and enriching the library fragments, the library was subjected to quality inspection. The library was paired-end (PE) sequenced with next-generation sequencing technology. The raw data were generated by the conversion of the sequencing platform’s own software. The clean data were obtained by filtering the raw data. We used the software TopHat2 HISAT2^1^ to align the clean reads to the reference genome (Kim et al., 2015). Reads aligned to intergenic regions (intergenic regions) may be transcribed from new genes or new noncoding RNAs.

The miRNA library was constructed by using TruSeq Small RNA Sample prep Kit (Illumina proprietary, RS-930-1012), and enriched by PCR amplification. The adapters were added at both ends of the miRNA. The miRNA library was purified by gel electrophoresis and quality checked by the Agilent High Sensitivity DNA Kit (Agilent, 5067-4626) using the Agilent 2100 Bioanalyzer. The miRNA library was quantified using the Quant-iT PicoGreen dsDNA Assay Kit (Invitrogen, P7589). And high-throughput sequencing was performed on the machine after determining the optimal loading amount of the miRNA library.

We analyzed gene expression using DESeq, and defined genes with | fold change| > 2, *p*-value < 0.05 as differentially expressed genes (Love et al., 2014). We used the R language ggplots2 software package to draw differentially expressed volcano diagrams. Transcript data were uploaded to the GEO database (GSE184660).

### 2.5. Construction of ceRNA regulatory networks

TargetScan and miRanda were used to predict lncRNAs, circRNAs, and mRNAs that have potential regulatory relationships with DE-miRNAs. We screened lncRNAs, circRNAs, and mRNAs from differentially expressed genes predicted to bind to DE-miRNAs with expression trends. Subsequently drawing networks regulatory networks based on the selected genes.

### 2.6. Analysis of GO and KEGG

We performed Gene Ontology (GO) and Kyoto Encyclopedia of Genes and Genomes (KEGG) analysis by using Blast2go and KAA, respectively. The enrichment analyses of differentially expressed genes or their parental or target genes using Metascape (Zhou et al., 2019). GO terms include molecular function (MF), biological process (BP) and cellular component (CC). We selected the top 10 GO terms with the lowest *p* value in each GO category to display. We selected the 20 most significantly enriched KEGG pathways for display.

### 2.7. Quantitative RT-PCR

We applied the miRcute Plus miRNA First-Strand cDNA Synthesis Kit (Tiangen, KR21) to reverse the total RNA and the miRcute Plus miRNA qPCR Detection Kit (SYBR Green; Tiangen, FP411) reagent to detect miRNA expression levels. We used HiScript II Q Select RT SuperMix for qPCR (+gDNA wiper; Vazyme, R223) to complete RNA reversal and used AceQ qPCR SYBR Green Master Mix (Vazyme, Q111) to quantify mRNA, lncRNA, and circRNA expression. The relative expression of genes was analyzed by the 2^−△△Ct^ method. U6 was an internal control of miRNAs (Fu et al., 2021; Woo et al., 2022). β-actin was internal control of mRNAs, lncRNAs, and circRNAs (Primer sequence listing is shown in Supplementary Table S7).

### 2.8. Statistical analysis

Data were presented as Mean ± SD and analyzed by GraphPad Prism 8.0 with unpaired Student’s t-test and the Tukey correction; *p* < 0.05, *p* < 0.01, *p* < 0.005 were defined as *, **, ***, respectively. And not significant was defined as ns.

## 3. Results

### 3.1. mRNA expression profile post H37Rv or H37Rv△1759c infection

**Figure 1:**
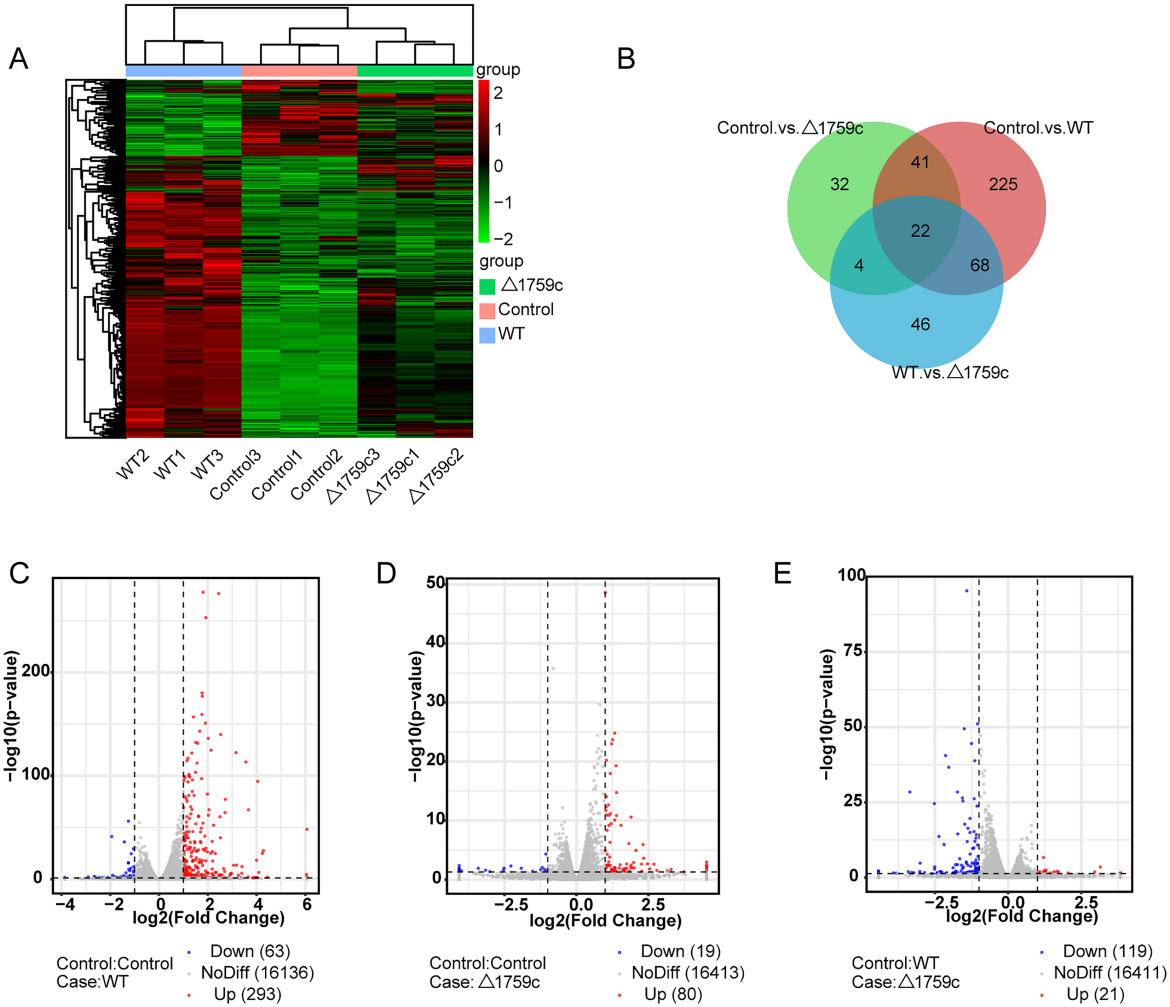
The mRNA expression profile of THP-1 cells after H37Rv or H37Rv△1759c infection. **(A)** A heatmap of all DE-mRNAs from the Control vs. WT group, Control vs. △1759c group, and WT vs. △1759c group. Each sample had three replicates (red: upward; green: downward). **(B)** Venn diagram of DE-mRNAs from the Control vs. WT group, Control vs. △1759c group, and WT vs. △1759c group. Volcano plot of the upregulated and downregulated DE-mRNAs from the Control vs. WT group **(C)**, Control vs. △1759c group **(D)**, and WT vs. △1759c group **(E)**.

THP1-derived-macrophages were able to control M. tb infection when inoculated with a low multiplicity of infection (MOI) of 1 (Welin et al., 2011). Our previous study showed that when H37Rv- and H37RvΔ1759c-infected macrophages at a MOI of 1, the anti-phagocytic capacity of H37RvΔ1759c was unchanged compared to H37Rv, but survival in macrophages was significantly reduced 24 h post infection (Dong et al., 2022). To investigate the mechanism by which macrophages control the infection of H37Rv at low MOI and the effect of Rv1759c on H37Rv survival, we collected THP-1 cells infected with H37Rv and H37Rv△1759c at MOI of 1 for 24 h for sequencing. Each treatment group contains three biological replicates. The uninfected cell group, H37Rv, and H37RvΔ1759c infection group was represented by Control, WT, and △1759c, respectively. We extracted the RNA of the collected samples, conducted quality inspection and qualified for subsequent library construction, sequencing and analysis. We used a clustering heatmap to display the expression of differentially expressed mRNAs (DE-mRNAs) post M. tb infection (Figure 1A). In the Control vs. WT group, Control vs. △1759c group, and WT vs. △1759c group, there were 356 DE-mRNAs (293 upregulated and 63 downregulated), 99 DE-mRNAs (80 upregulated and 19 downregulated), and 140 DE-mRNAs (21 upregulated and 119 downregulated; Supplementary Table S1), respectively. We described these DE-mRNAs using volcano diagrams (Figures 1C–E). We used a Venn diagram to present the distribution of DE-mRNAs in comparison groups (Figure 1B). From the Venn diagram, we found 90 DE-mRNAs associated with Rv1759c. There were 22 DE-mRNAs appeared simultaneously in the three comparison groups, indicating that Rv1759c co-regulates their expression with other components of H37Rv. The deletion of Rv1759c reduces the stimulation of H37Rv on the expression of these 22 DE-mRNAs (including CXCL2, PTGS2, CXCL1, IL-23A, IFIT2, IFIT3, IFIT1, and CXCL11). There were 68 DE-mRNAs appeared in the Control vs. WT group and the WT vs. △1759c group, but not in the Control vs. △1759c group. The altered expression of these 68 DE-mRNAs (including CAMKV, SOD2, NFKBIZ, CCR7, IL6, IL36G, and CXCL13) was only caused by stimulation of Rv1759c.

**Figure 2:**
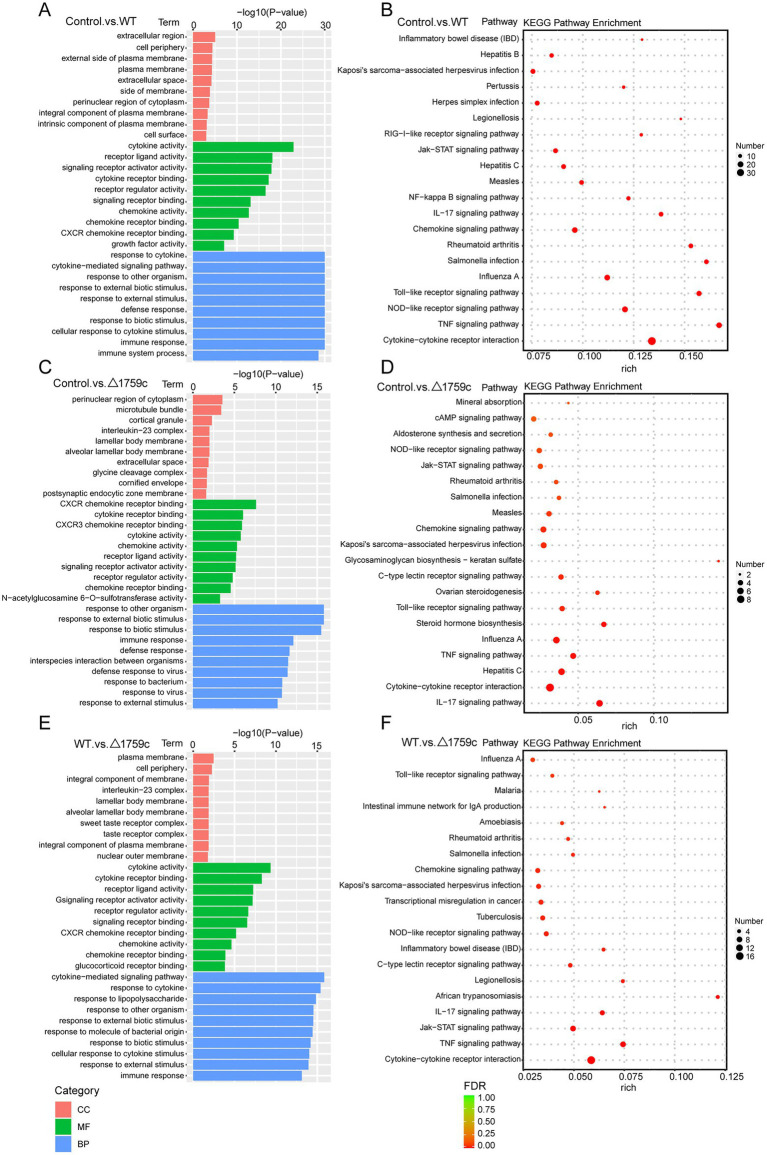
GO and KEGG pathway analysis for the DE-mRNAs after H37Rv or H37Rv△1759c infection. The GO enrichment analysis results of DE-mRNAs from the Control vs. WT group **(A)**, Control vs. △1759c group **(C)**, and WT vs. △1759c group **(E)**. The 10 most significantly enriched GO terms in each GO category are selected for display. KEGG enrichment analysis results of DE-mRNAs from Control vs. WT group **(B)**, Control vs. △1759c group **(D)**, and WT vs. △1759c group **(F)**, and the top 20 KEGG pathways with the smallest *p*-value are selected for presentation.

**Figure 3:**
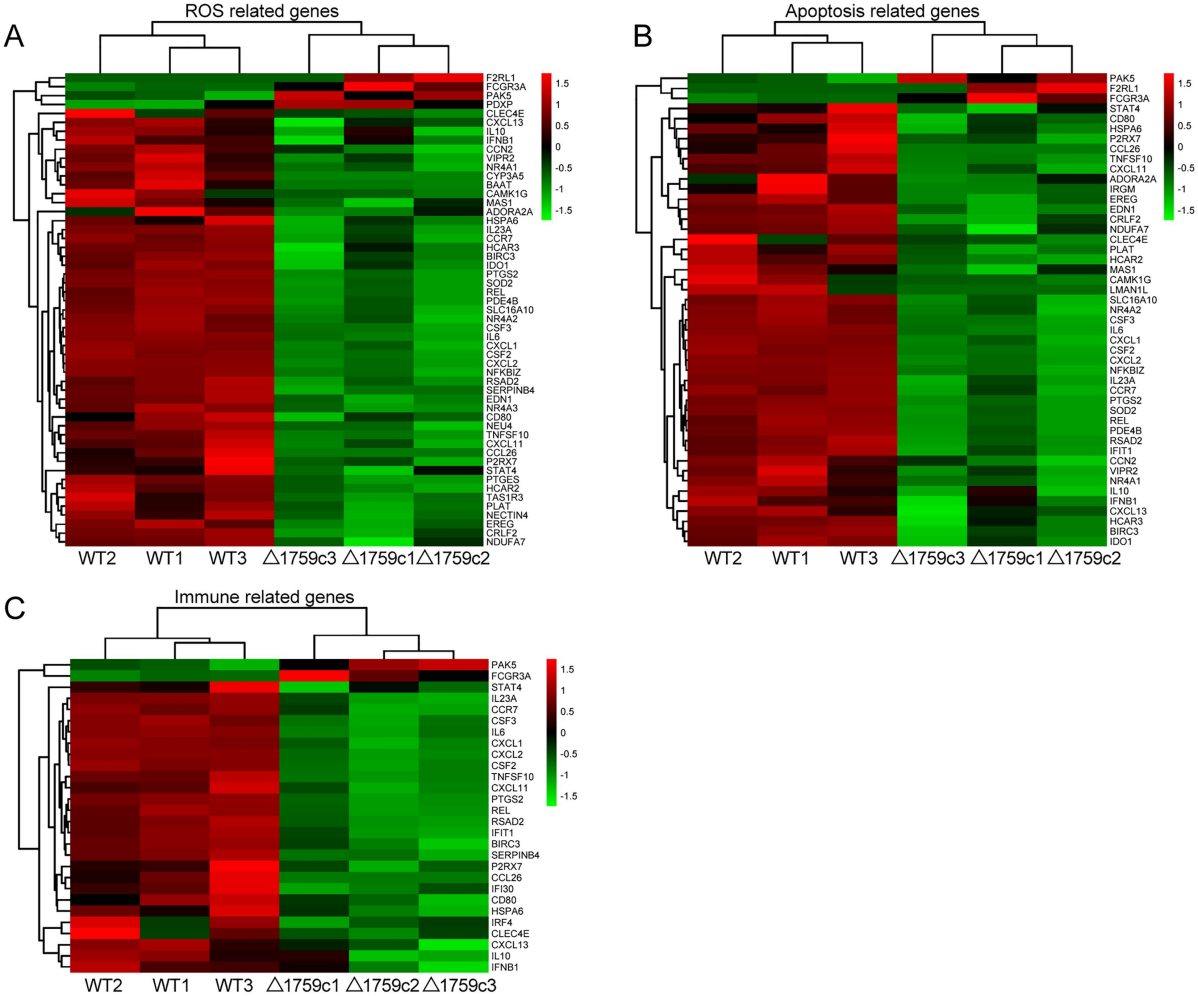
Cluster analysis of DE-mRNAs associated with ROS production, apoptosis, and immunity affected by Rv1759c. **(A)** A heatmap of 53 DE-mRNAs from WT vs. △1759c group related to ROS production. **(B)** A heatmap of 49 DE-mRNAs from WT vs. △1759c group related to apoptosis. **(C)** A heatmap of 28 DE-mRNAs from WT vs. △1759c group related to immunity. Each sample had three replicates (red: upward; green: downward).

We performed gene ontology (GO) and KEGG enrichment analysis for these DE-mRNAs (Supplementary Table S1). GO enrichment analysis divides genes into cell component (CC), molecular function (MF), and biological process (BP). We selected the top 10 GO terms and the top 20 KEGG pathways with shown significant enrichment. We found that DE-mRNAs in the three comparison groups were mostly enriched in the same GO terms and KEGG pathways, such as cytokine activity and chemokine activity in MF; immune response in BP; and cytokine-cytokine receptor interaction, chemokine signaling pathway, TNF signaling pathway, and IL-17 signaling pathway. These results are consistent with previous literatures mentioned the pathways by which M. tb activates macrophages inflammatory response (Flynn and Chan, 2001; van Crevel et al., 2002; Cooper and Khader, 2008). But the significance degree of enrichment to the GO terms and the number of DE-mRNAs enriched to the KEGG pathway varied. During H37Rv infection, the degree of significance enriched to GO terms was highest and the number of DE-mRNAs enriched to KEGG pathways was highest, which decreased after Rv1759c deletion. The results of GO and KEGG analysis in the WT vs. △1759c group were similar to the results in Control vs. WT group and the Control vs. △1759c group. The above results may be due to that the deletion of Rv1759c gene changed the M. tb stimulation intensity and activation levels of different signaling pathway. There are also some signaling pathways that are only enriched in the Control vs. WT group but not in the Control vs. △1759c group, such as the NF-κB signaling pathway (Figure 2). In addition, we selected 53, 49, and 28 DE-mRNAs in the WT vs. Δ1759c group to draw heatmaps related to ROS generation, apoptosis and immunity, respectively. The results showed that the expression of genes related to these pathways was different, suggesting that the role of Rv1759c in the process of M. tb infection may be caused by affecting these pathways (Figure 3; Supplementary Table S8).

### 3.2. lncRNA expression profile post H37Rv or H37Rv△1759c infection

**Figure 4:**
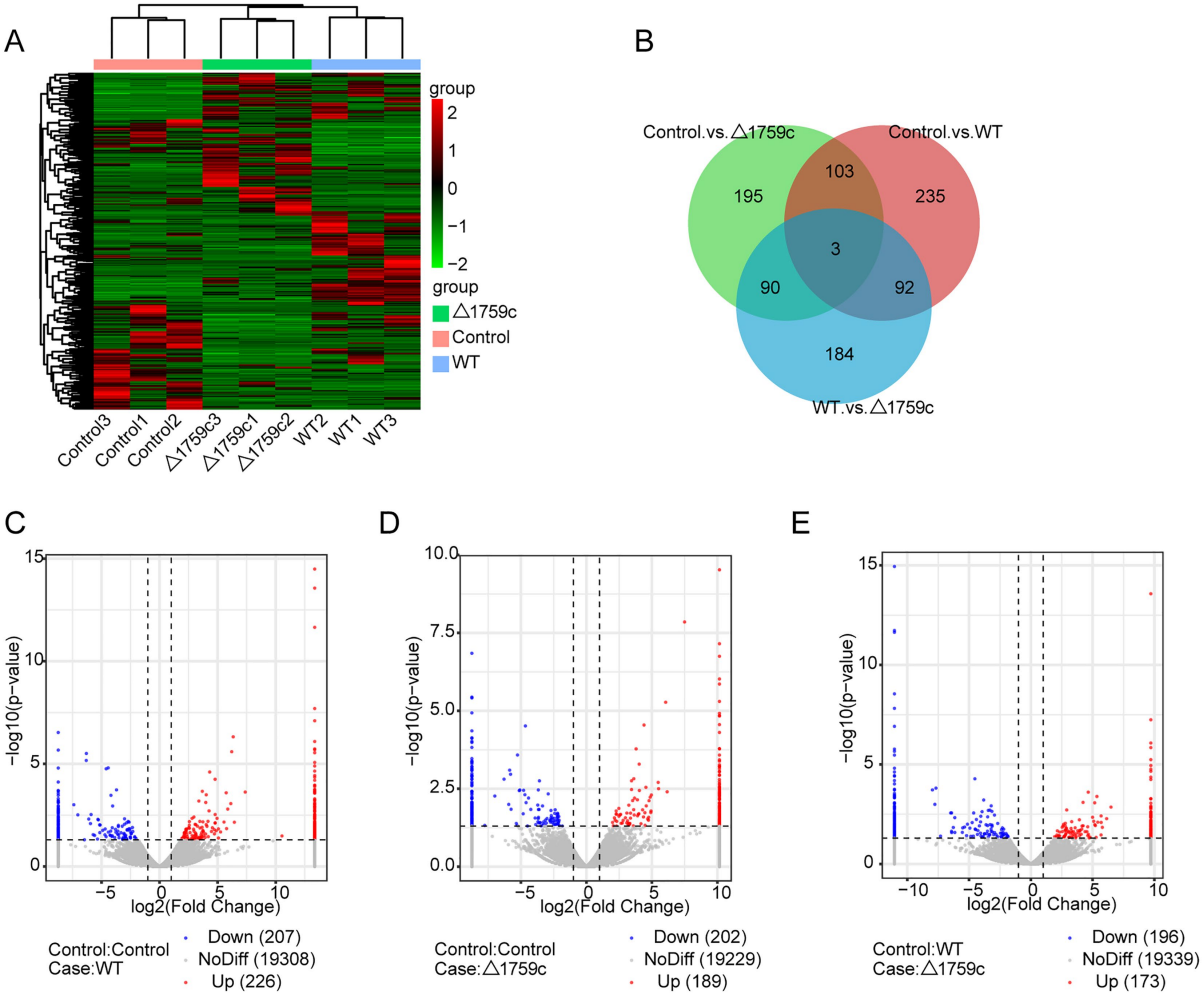
The lncRNA expression profile of THP-1 cells after H37Rv or H37Rv△1759c infection. **(A)** A heatmap of all DE-lncRNAs from the Control vs. WT group, Control vs. △1759c group, and WT vs. △1759c group. Each sample had three replicates (red: upward; green: downward). **(B)** Venn diagram of DE-lncRNAs from the Control vs. WT group, Control vs. △1759c group, and WT vs. △1759c group. Volcano plot of the increased and decreased DE-lncRNAs from the Control vs. WT group **(C)**, Control vs. △1759c group **(D)** and WT vs. △1759c group **(E)**.

Previous studies demonstrated that lncRNAs can play their function through a variety of mechanisms. Subcellular localization of lncRNAs determines how they function (Bridges et al., 2021). The lncRNAs localized in the nucleus can interact with proteins and regulate transcriptional or epigenetic (Saxena and Carninci, 2011; Melé and Rinn, 2016); lncRNAs localized in the cytoplasm can play roles through post-transcriptional regulation. LncRNAs can bind to miRNA as ceRNA and regulate the target genes expression (Wang et al., 2017; Yang et al., 2020), or mediate mRNA translation and stability (Yuan et al., 2017). And lncRNAs recruited to ribosomes may function by being translated into small peptides (Huang et al., 2017; Wang et al., 2020). LncRNAs are located upstream or downstream of protein-coding genes and affect their expression through transcription or translation (Joung et al., 2017). We spliced the obtained sequencing data and compare with the human reference genome for lncRNA screening. We screened candidate lncRNA transcripts which meeting the following conditions: the length of transcripts is greater than 200 bp and more than or equal to 2 exons; transcripts are one of anti-sense lncRNA, intergenic lncRNA, or intronic lncRNA types; transcripts that appeared at least 3 times in one sample. Subsequently, we screened a total of 1,022 novel lncRNA transcripts with high confidence that were identified by PLEK, CNCI, and Pfamscan with no coding potential. The expressions of lncRNAs in H37Rv, △1759c, and Control were compared and analyzed. There were 433 DE-lncRNAs (226 upregulated and 207 downregulated) in the Control vs. WT group, 391 DE-lncRNAs (189 upregulated and 202 downregulated) in the Control vs. △1759c group, and 369 DE-lncRNAs (173 upregulated and 196 downregulated) in the WT vs. △1759c group. A total of 1,022 novel lncRNAs were identified, of which 93 were DE-lncRNAs in the Control vs. WT group, and 56 were DE-lncRNAs in the Control vs. △1759c group, and 63 were DE-lncRNAs in the WT vs. △1759c group (Supplementary Table S2). The clustering heatmap was used to present the expression of DE-lncRNAs post M.tb infection (Figure 4A). The distribution of DE-lncRNAs is shown in the Venn diagram (Figure 4B), and the DE-lncRNAs in different compared groups are represented by volcano diagrams (Figures 4C–E). We found that 3 DE-lncRNAs (ENST00000418273, two novel lncRNAs MSTRG.6115.6, MSTRG.770.18) appeared simultaneously in the three comparison groups, which co-regulated by Rv1759c and other genes. And 92 DE-lncRNAs appeared in the Control vs. WT group and WT vs. △1759c group, but not in the Control vs. △1759c group, which only regulated by Rv1759c. These results indicate that Rv1759c affects numerous lncRNAs expression.

**Figure 5:**
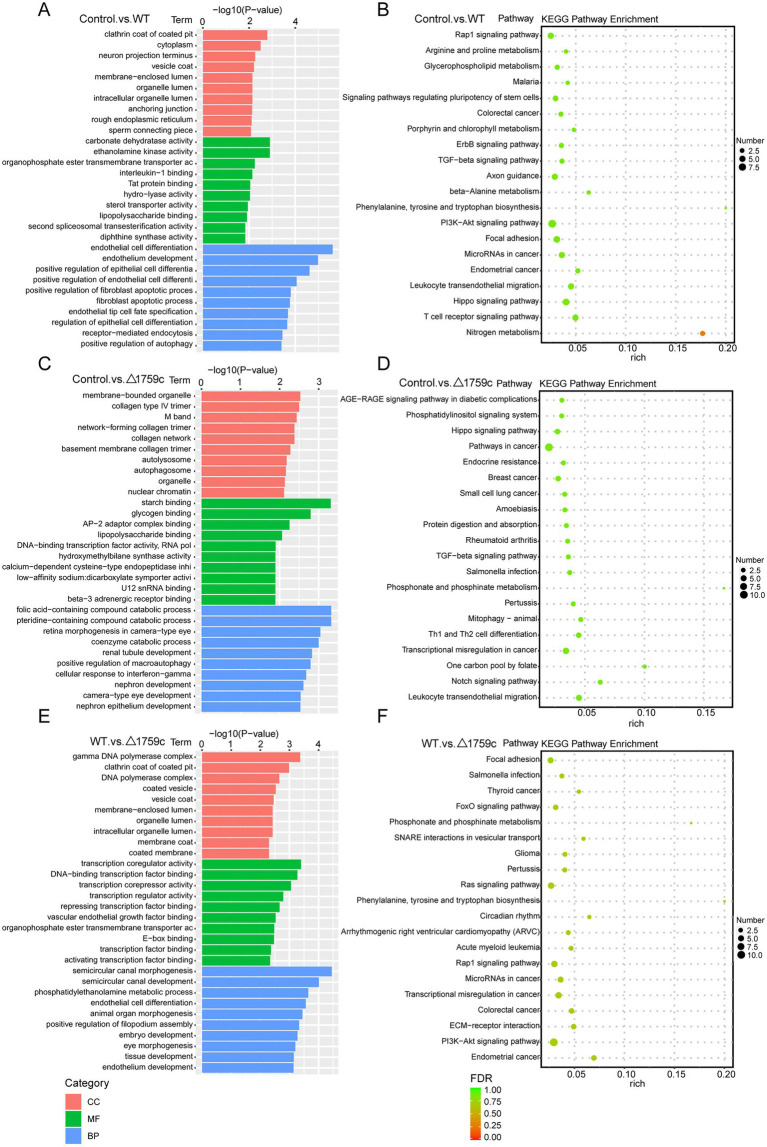
GO and KEGG pathway analysis for DE-lncRNAs target genes after H37Rv or H37Rv△1759c infection. The GO enrichment analysis results of target genes of DE-lncRNAs from the Control vs. WT group **(A)**, Control vs. △1759c group **(C)**, and WT vs. △1759c group **(E)**. The 10 most significantly enriched GO terms in each GO category are selected for display. KEGG enrichment analysis results of target genes of DE-lncRNAs from the Control vs. WT group **(B)**, Control vs. △1759c group **(D)**, and WT vs. △1759c group **(F)**, and the top 20 KEGG pathways with the smallest *p*-value are selected for presentation.

We annotated and enriched analyses lncRNA target mRNAs (Supplementary Table S2). GO analysis showed that the GO terms enriched for DE-lncRNAs target genes in different comparison groups were quite different. In the Control vs. WT group, DE-lncRNAs target genes were observably concentrated in positive regulation of autophagy in BP category (Figure 5A). In the WT vs. △1759c group, DE-lncRNAs target genes influenced by Rv1759c concentrated in transcription regulator activity in MF category. We found that DE-mRNAs in Control vs. WT group and WT vs. △1759c group but not in Control vs. △1759c group were enriched in Rap1 signaling pathway, focal adhesion, and PI3K-Akt signaling pathway. It was reported that Rap1 can activate downstream inflammatory responses through the NF-κB signaling pathway or Toll-like receptors (Tang et al., 2014; Zhang et al., 2015). The PI3K-Akt signaling pathway is involved in autophagy and inflammatory response-related signaling pathways during M. tb infection (Bai et al., 2014; Liu et al., 2016; Shariq et al., 2021). These results suggest that genes targeted by DE-lncRNAs affected by Rv1759c may be involved in autophagy and inflammatory responses during M. tb infection through these signaling pathways (Figure 5).

### 3.3. circRNA expression profile post H37Rv or H37Rv△1759c infection

**Figure 6:**
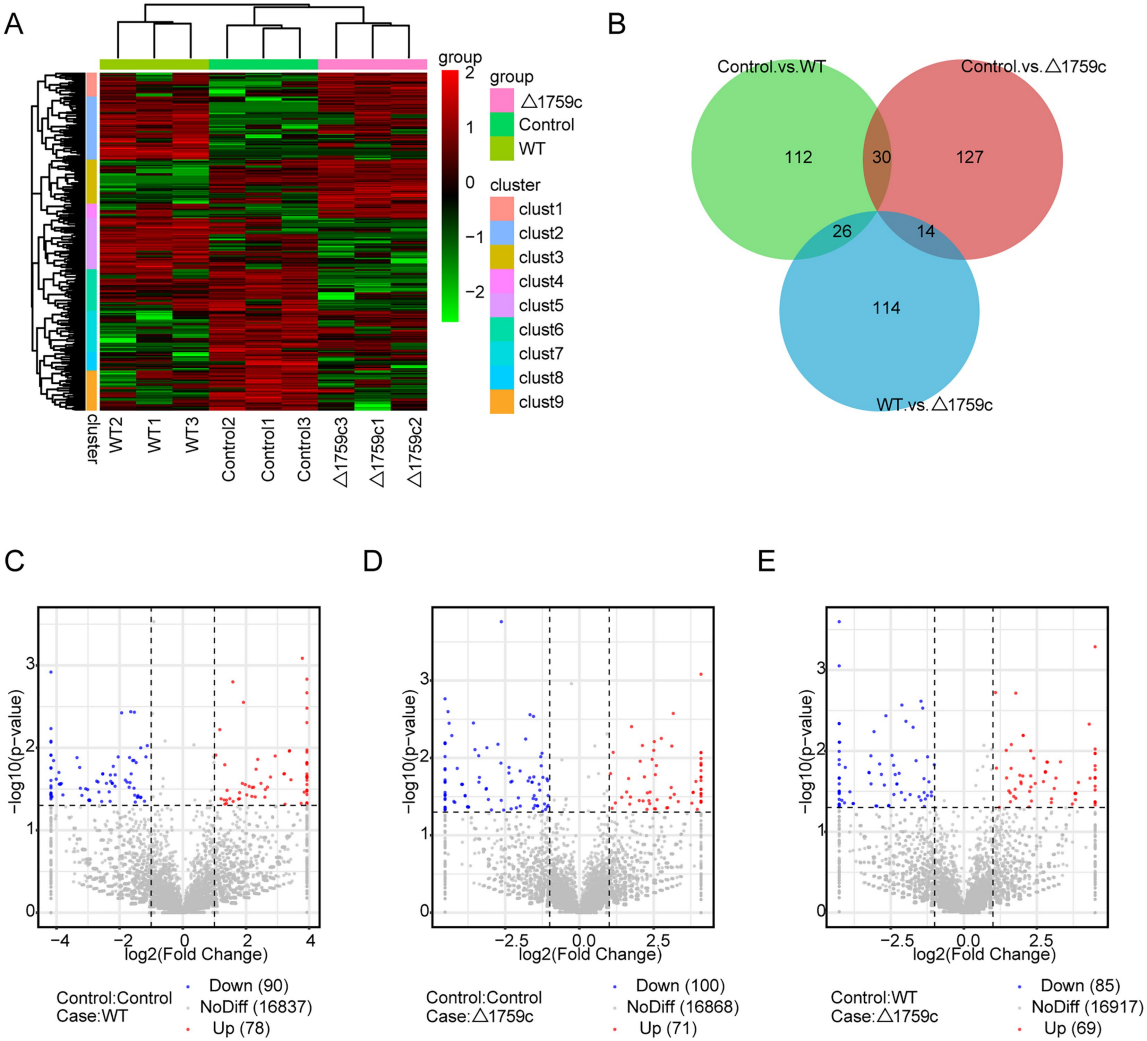
The circRNA expression profile of THP-1 cells after H37Rv or H37Rv△1759c infection. **(A)** A heatmap of all DE-circRNAs from the Control vs. WT group, Control vs. △1759c group, and WT vs. △1759c group. Each sample had three replicates (red: upward; green: downward). **(B)** Venn diagram of DE-circRNAs from the Control vs. WT group, Control vs. △1759c group, and WT vs. △1759c group. Volcano plot of the up and down -regulated DE-circRNAs from the Control vs. WT group **(C)**, Control vs. △1759c group **(D)** and WT vs. △1759c group **(E)**.

We compared the anchor sequence with the reference genome and screened out circRNAs with high reliability. We classified and annotated the circRNA according to results identified by find_circ and the annotation information of the genome. There were 168 DE-circRNAs (78 upregulated and 90 downregulated), 171 DE-circRNAs (71 upregulated and 100 downregulated) and 154 DE-circRNAs (69 upregulated and 85 downregulated) in the Control vs. WT group, Control vs. △1759c group, and WT vs. △1759c group, respectively (Supplementary Table S3). We used a clustering heatmap to show the expression profile of circRNAs post M. tb infection (Figure 6A), and sub-clustered DE-circRNAs into different clusters according to their expression patterns (DE-circRNAs in the same cluster have similar expression trends; Supplementary Table S3). The distribution of DE-circRNAs is shown in the Venn diagram (Figure 6B), and the DE-circRNAs in different compared groups are represented by volcano diagrams (Figures 6C–E). The distribution of DE-circRNAs in the three comparison groups was not completely consistent with the distribution of DE-mRNAs and DE-lncRNAs. There is no DE-circRNA differentially expressed in the three comparison groups, only 26 DE-circRNAs in Control vs. WT and WT vs. △1759c group. This indicates the 26 DE-circRNAs are only related to the deletion of Rv1759c, which is relatively less compared with the number of DE-lncRNAs.

**Figure 7:**
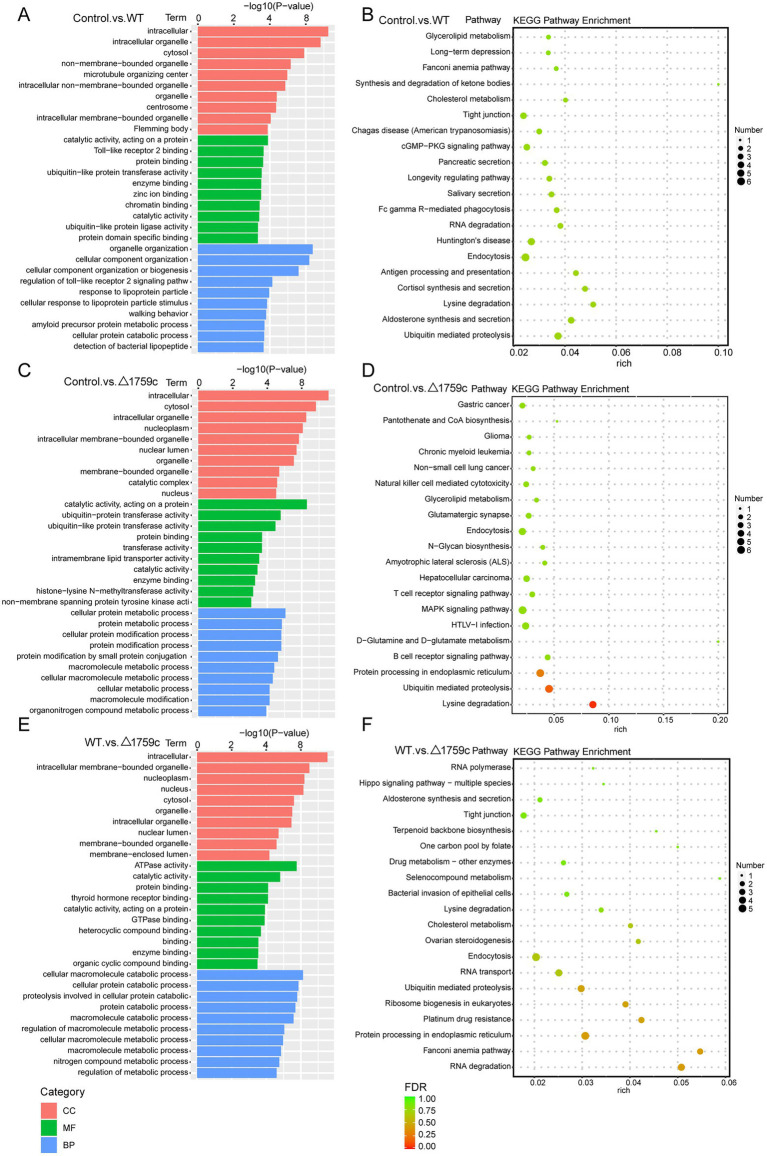
GO and KEGG pathway analysis for DE-circRNAs parental genes after H37Rv or H37Rv△1759c infection. The GO enrichment analysis results of parental genes of DE-circRNAs from the Control vs. WT group **(A)**, Control vs. △1759c group **(C)**, and WT vs. △1759c group **(E)**. The 10 most significantly enriched GO terms in each GO category are selected for display. KEGG enrichment analysis results of parental genes of DE-circRNAs from Control vs. WT group **(B)**, Control vs. △1759c group **(D)**, and WT vs. △1759c group **(F)**, and the top 20 KEGG pathways with the smallest *p*-value are selected for presentation.

We performed GO and KEGG enrichment analysis for the DE-circRNA parental genes (Supplementary Table S3). The DE-circRNA parental genes in the three comparison groups were most obviously enriched intracellular GO term in the CC category. This is different from the enrichment results of DE-lncRNAs target genes, which are more enriched in GO terms belonging to MF and BP categories. It suggests that noncoding RNAs may have preferences for regulating different signaling pathways in the process of M. tb infection. The results of KEGG analysis showed that DE-circRNA parental genes in the three comparison groups were enriched in ubiquitin mediated proteolysis pathway and endocytosis pathway which were demonstrated in previous literature to regulate M. tb survival in macrophages (Pieters, 2008; Wang J. et al., 2015; Franco et al., 2017; Liu et al., 2017; Yang and Ge, 2018; Chai et al., 2019). It is suggested that DE-circRNA parental genes which were agminated in ubiquitin mediated proteolysis and endocytosis during H37Rv and H37Rv△1759c infection are involved in M. tb survival. This result also suggests that Rv1759c regulated DE-circRNAs parental genes involved in the activation of endocytosis and ubiquitination pathways post M. tb infection (Figure 7).

### 3.4. miRNA expression profile post H37Rv or H37Rv△1759c infection

**Figure 8:**
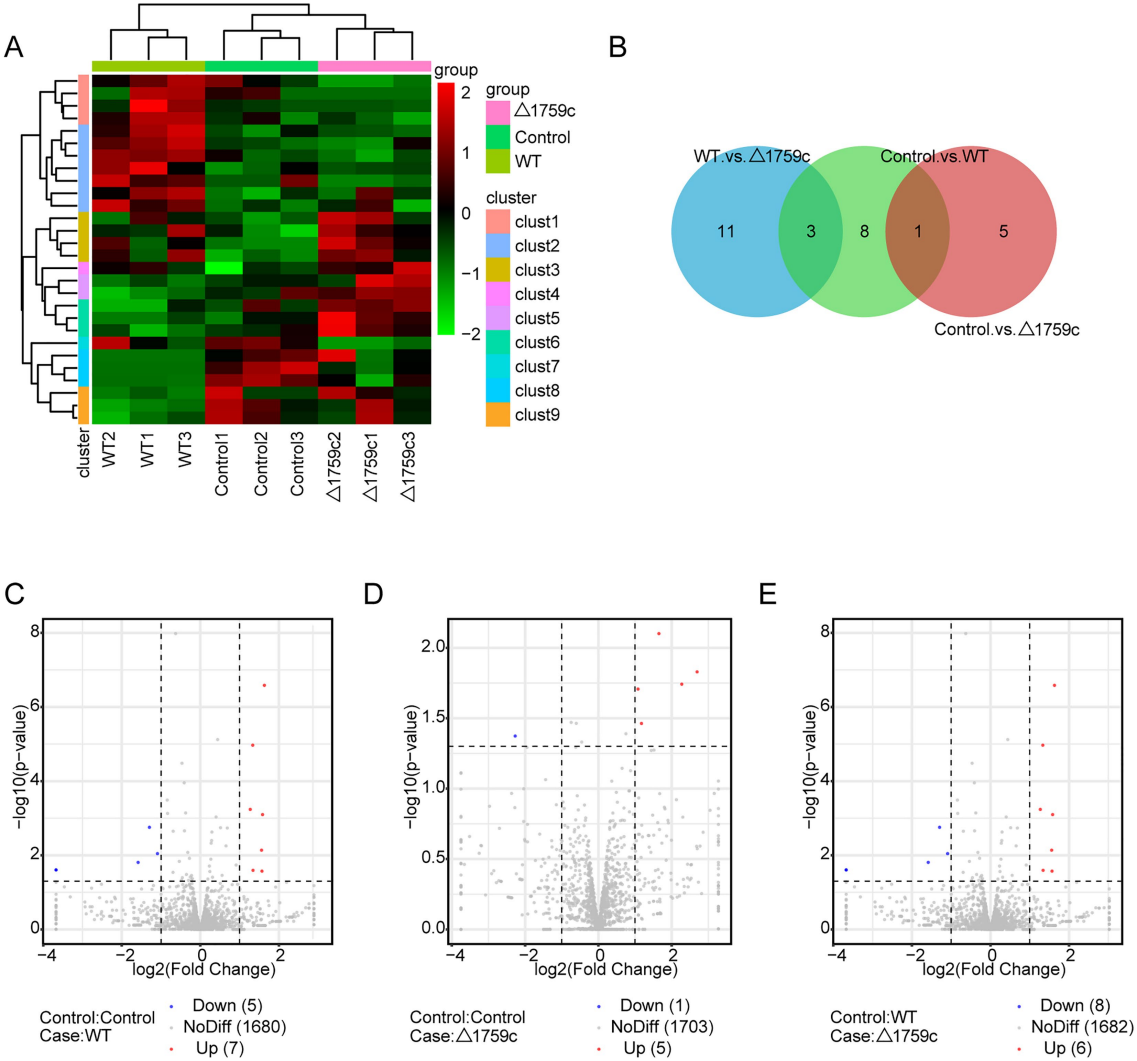
The miRNA expression profile of THP-1 cells after H37Rv or H37Rv△1759c infection. **(A)** A heatmap of all DE-miRNAs from the Control vs. WT group, Control vs. △1759c group, and WT vs. △1759c group. Each sample had three replicates (red: upward; green: downward). **(B)** Venn diagram of DE-miRNAs from the Control vs. WT group, Control vs. △1759c group, and WT vs. △1759c group. Volcano plot of the increased and decreased DE-miRNAs from the Control vs. WT group **(C)**, Control vs. △1759c group **(D)** and WT vs. △1759c group **(E)**.

We constructed the miRNA library, which was sequenced after purification and quality inspection. The sequencing data were compared with the human reference genome for annotating and obtaining abundance information. There were 12 DE-miRNAs (7 upregulated and 5 downregulated), 6 DE-miRNAs (5 upregulated and 1 downregulated), and 14 DE-miRNAs (6 upregulated and 8 downregulated) in the Control vs. WT group, Control vs. △1759c group, and WT vs. △1759c group, respectively (Supplementary Table S4). We used a clustering heatmap to show the expression profile of miRNAs post M. tb infection and sub-clustered DE-miRNAs into different clusters according to their expression patterns (DE-miRNAs in the same cluster have similar expression trends; Supplementary Table S4; Figure 8A). The distribution of DE-miRNAs is shown in the Venn diagram (Figure 8B), and the DE-miRNAs in different compared groups are represented by volcano diagrams (Figures 8C–E). We found that the three DE miRNAs (hsa-miR-12136, hsa-miR-4485-3p, and hsa-miR-7704) appeared in WT vs.△1759c group and Control vs. WT group, and their expression changes were only related to Rv1759c.

**Figure 9:**
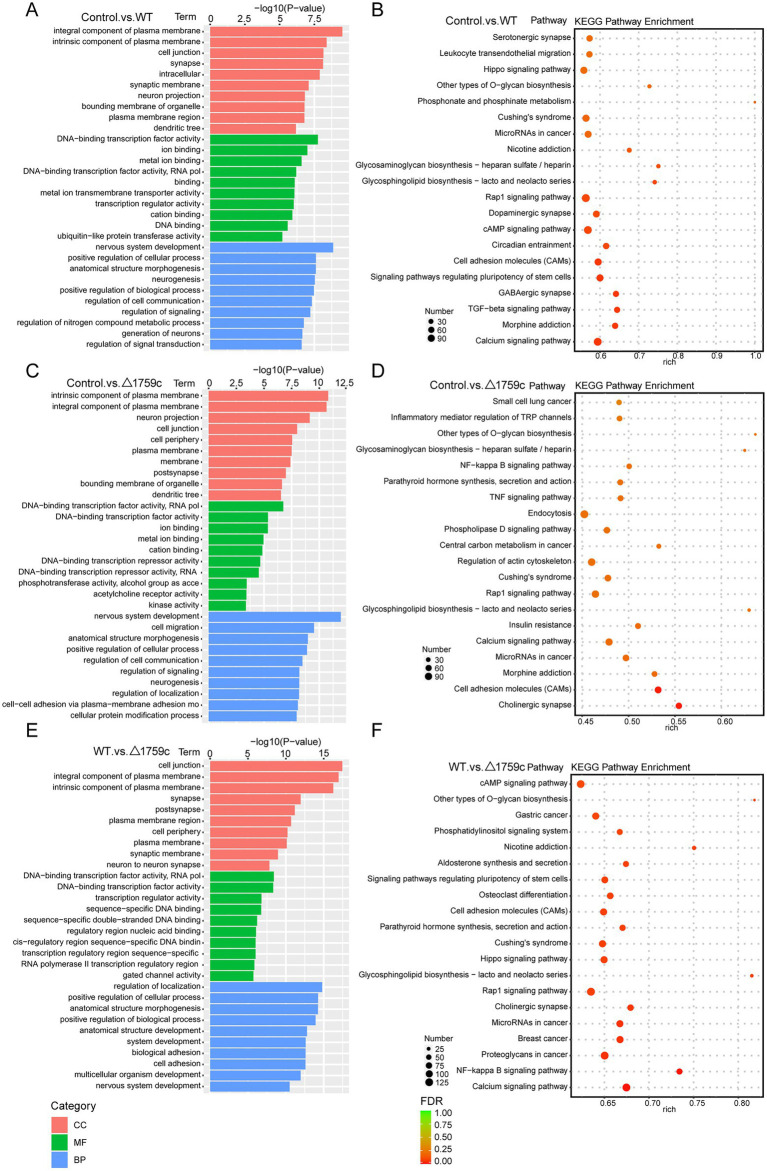
GO and KEGG pathway analysis for the target genes of DE-miRNAs after H37Rv or H37Rv△1759c infection. The GO enrichment analysis results of target genes of DE-miRNAs from the Control vs. WT group **(A)**, Control vs. △1759c group **(C)**, and WT vs. △1759c group **(E)**. The 10 most significantly enriched GO terms in each GO category are selected for display. KEGG enrichment analysis results of target genes of DE-miRNAs from Control vs. WT group **(B)**, Control vs. △1759c group **(D)**, and WT vs. △1759c group **(F)**, and the top 20 KEGG pathways with the smallest *p*-value are selected for presentation.

MiRNAs mainly exert their regulatory function through targeting the UTR region of mRNAs (Xie et al., 2005; Ransohoff et al., 2018). The target genes of DE-miRNAs were predicted by TargetScan and RNAhybrid and performed GO and KEGG pathway analysis (Supplementary Table S4). It was found that DE-miRNAs target genes in the three comparison groups were enriched in DNA-binding transcription factor activity GO term of MF category. This suggested that the target genes of DE-miRNAs affected by Rv1759c may function through DNA-binding transcription factors during M. tb infection. KEGG pathway analysis result showed that calcium-signaling pathway was enriched in the three compared groups, consistent with the results described in the previous literatures (Ouimet et al., 2016; Liu et al., 2018). The NF-κB signaling pathway was enriched in the Control vs. △1759c group and WT vs. △1759c group, suggesting that DE-miRNAs target genes affected by Rv1759c are correlated with the NF-κB signaling pathway (Figure 9).

### 3.5. Verification of differentially expressed genes

We technically replicated the results of the sequencing using qPCR. We randomly selected 3 DE-mRNAs (CCL20, CXCL20, and IFIT1), 3 DE-miRNAs (hsa-miR-1268a hsa-miR-1271-5p and hsa-miR-4638-5p), 3 DE-lncRNAs (ENST00000647388, ENST00000445682, and ENST00000666623) and 3 DE-circRNAs (hsacirc_036051, hsacirc_041934, and hsacirc_065432) and verified their expression in 9 samples by qPCR (Supplementary Figures S1A,C,E,G). The qPCR results and RNA-Seq data were consistent (Supplementary Figures S1B,D,F,H).

### 3.6. ceRNA networks during H37Rv infection

**Figure 10:**
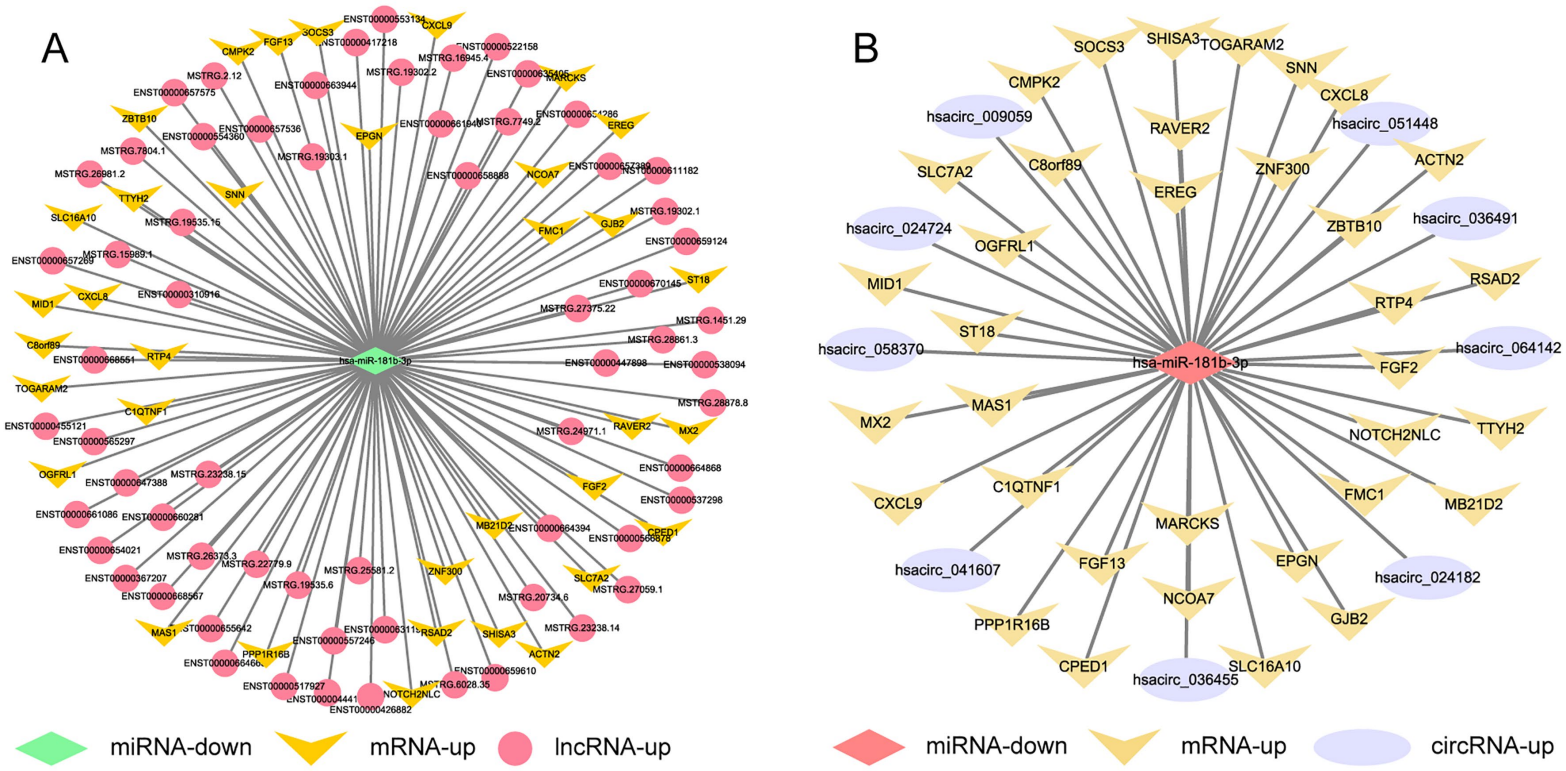
Competing endogenous RNA regulatory networks centered on hsa-miR-181b-3p post H37Rv infection. **(A)** The lncRNA-miR-181b-3p-mRNA regulation network after H37Rv infection. **(B)** The circRNA-miR-181b-3p -mRNA regulation network after H37Rv infection.

**Figure 11:**
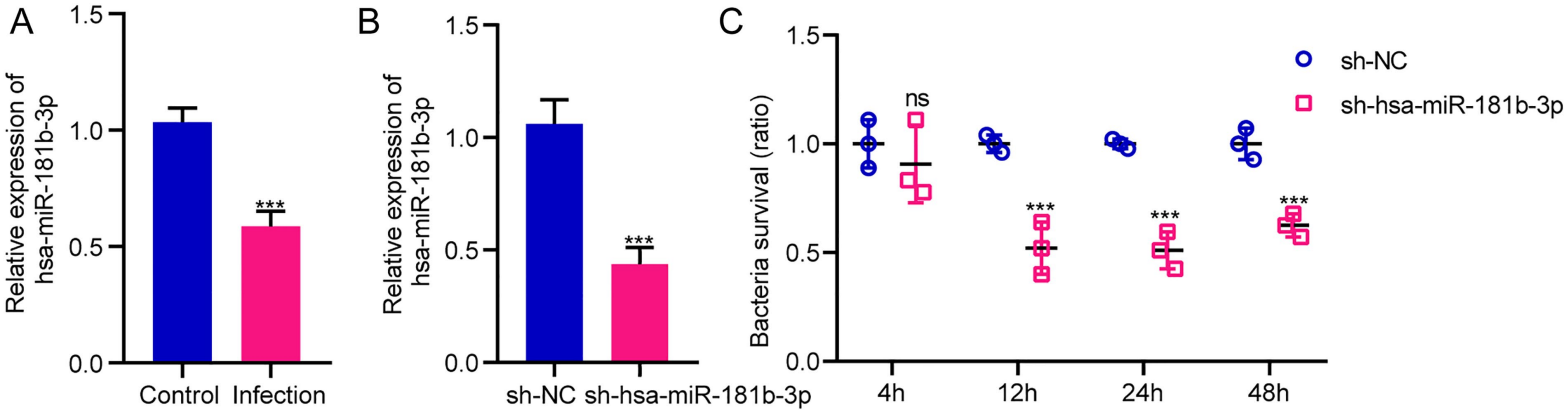
Silencing hsa-miR-181b-3p reduces intracellular survival of H37Rv. **(A)** Quantitative real-time PCR (qRT-PCR) detection of the expression of hsa-miR-181b-3p in THP-1 cells of the control group and cells infected with H37Rv. **(B)** qRT-PCR detection of the expression of hsa-miR-181b-3p in sh-NC THP-1 and sh-hsa-miR-181b-3p THP-1 cells. **(C)** sh-NC THP-1 and sh-hsa-miR-181b-3p THP-1 cells were infected with H37Rv and then subjected to CFU analysis.

The sequencing results of H37Rv-infected macrophages showed a new miRNA transcriptional expression profile. The function of these DE-miRNAs during H37Rv infection remains unreported. To explore the function of these miRNAs and the regulatory roles of lncRNAs or circRNAs as ceRNAs during M. tb infection, we screened the lncRNAs, circRNAs, and mRNAs predicted to interact with DE-miRNAs from the differentially expressed genes in the Control vs. WT group based on the negative regulation patterns and software prediction results, and then constructed the lncRNA/circRNA-miRNA-mRNA regulatory networks centered on the DE-miRNAs during H37Rv infection. There were 45 lncRNAs, 9 circRNAs, and 8 mRNAs that had a regulatory relationship with miR-129-1-3p. There were 99 lncRNAs, 22 circRNAs, and 7 mRNAs interacting with miR-6772-3p. There were 64 lncRNAs, 9 circRNAs, and 35 mRNAs that could bind to miR-181b-3p. There were 4 DE-circRNAs targeting hsa-miR-135b-3p that regulated 5 DE-mRNAs (Supplementary Table S5). We used miR-129-1-3p, miR-6772-3p and miR-181b-3p as the center to generate the lncRNA-miRNA-mRNA network diagram (Supplementary Figure S2A). We used miR-129-1-3p, miR-6772-3p, miR-135b-3p, and miR-181b-3p as the center to generate the circRNA-miRNA-mRNA network diagram (Supplementary Figure S2B). There were 25 DE-lncRNAs that interacted with miRNA-1291-3p and miR-6772-3p, and the common target DE-mRNAs were TLR4 and BSN. There were no DE-circRNAs that interacted with miR-129-1-3p, miR-6772-3p, and miR-135b-3p, but there were DE-circRNAs that had a regulatory relationship with two of them. We selected hsa-miR-181b-3p, the miRNA with the largest fold change, to draw the network diagrams for easy information acquisition (Figure 10). We re-collected the samples of H37Rv-infected THP-1 cells to verify the expression of the network center molecule hsa-miR-181b-3p, and the results showed that hsa-miR-181b-3p expression was significantly downregulated (Figure 11A). To verify the effect of hsa-miR-181b-3p on the survival of H37Rv in macrophages, we constructed sh-hsa-miR-181b-3p THP-1 cell line and confirmed the hsa-miR-181b-3p expression by qPCR. The expression of hsa-miR-181b-3p was significantly decreased in hsa-miR-181b-3p THP-1 cells compared with sh-NC THP-1 cells (Figure 11B). Subsequently, we infected sh-NC THP-1 and hsa-miR-181b-3p THP-1 cells with H37Rv, and detected the survival of H37Rv at 4, 12, 24, and 48 h post infection. The results showed that silencing hsa-miR-181b-3p can significantly reduce the survival of H37Rv in THP1 derived macrophages (Figure 11C). Our results demonstrate that the hub of the non-coding RNA network influences the survival of M. tb.

### 3.7. ceRNA networks post H37Rv△1759c infection

**Figure 12:**
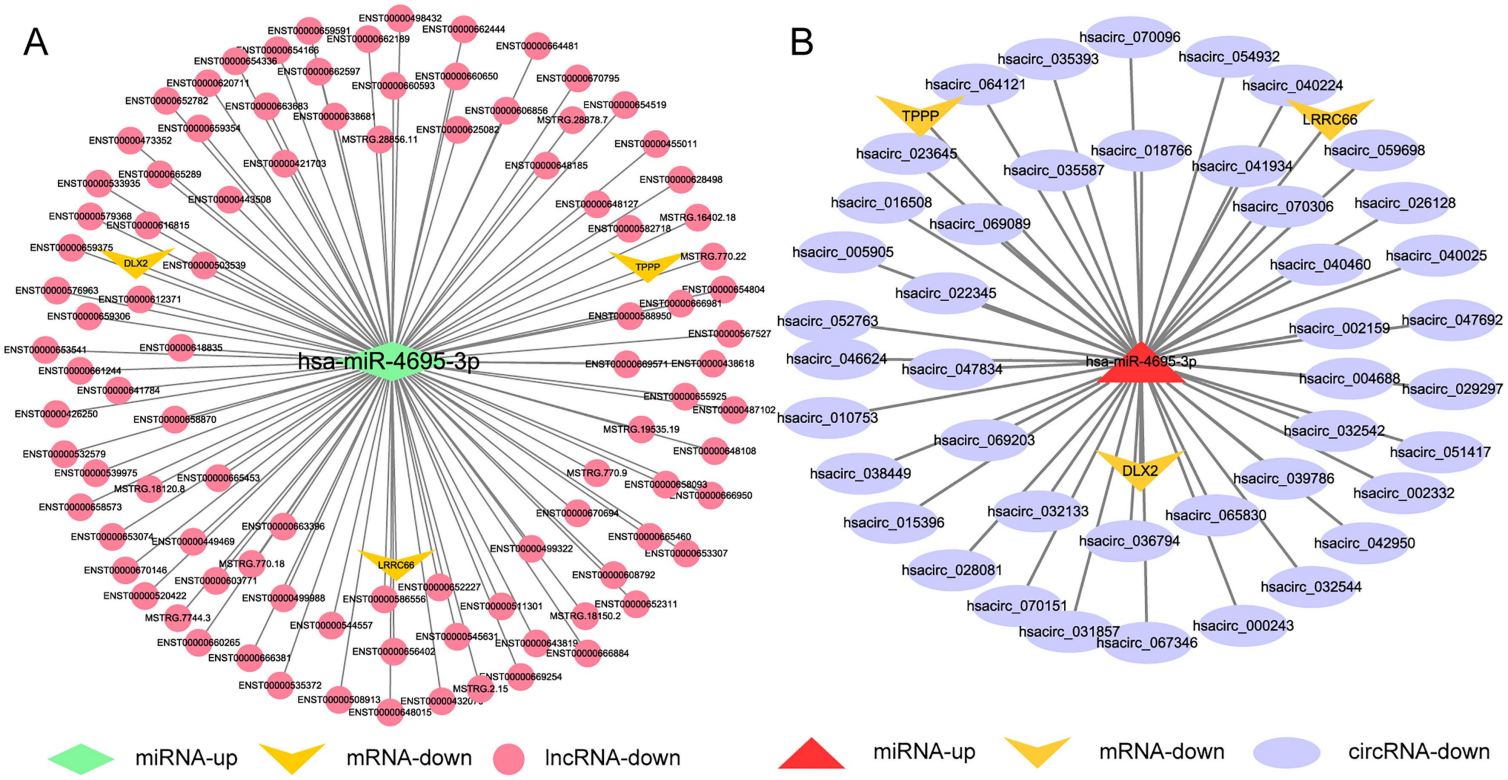
Competing endogenous RNA regulatory networks centered on hsa-miR-4695-3p post H37Rv△1759c infection. **(A)** The lncRNA-miR-4695-3p-mRNA regulation network after H37Rv△1759c infection. **(B)** The circRNA-miR-4695-3p-mRNA regulation network after H37Rv△1759c infection.

We used the same method as above to screen out the lncRNAs, circRNAs, and mRNAs from the differentially expressed genes that are predicted to bind to DE-miRNAs during H37Rv△1759c infection and constitute the ceRNA networks diagram. There were 89 lncRNAs, 26 circRNAs, and 1 mRNA that interacted with hsa-miR-6772-3p. There were 96 lncRNAs, 43 circRNAs, and 3mRNAs that interacted with hsa-miR-4695-3p. There were 33 lncRNAs, 43 circRNAs, and 6 mRNAs that interacted with hsa-miR-6511a-5p. There were 99 lncRNAs, 38 circRNAs, and 3 mRNAs that interacted with hsa-miR-1291. There were 66 lncRNAs, 29 circRNAs, and 3 mRNAs that interacted with hsa-miR-6774-5p. There were 88 lncRNAs, 24 circRNAs, and 2 mRNAs that interacted with hsa-miR-1271-5p (Supplementary Table S6). Centering on these DE-miRNAs, we constructed non-coding RNA interactions networks (Supplementary Figures S3A,B). We selected the largest fold change miRNA, hsa-miR-4695-3p, to draw the network diagrams for easy information acquisition (Figure 12). In the network diagram, 17 decreased DE-lncRNAs and 5 decreased DE-circRNAs co-regulated 3 upregulated DE-miRNAs. There were 3 DE-miRNAs co-targeted TPPP and XKR7, and 2 DE-miRNAs targeted TUBB8, DLX2, and LRRC66. The results show that the regulatory mechanism of the expression of these DE-mRNAs is complicated. The literature shows that TPPP can impair autophagosome-lysosome fusion (Ejlerskov et al., 2013). The function of TPPP in M. tb has not been reported, which may be regulate the process of autophagy during M. tb infection. Studies have shown that DLX2 is involved in apoptosis, and increased apoptosis can promote the elimination of M. tb (Yilmaz et al., 2011; Dai et al., 2013; Raja et al., 2017). Whether DLX2 regulates the apoptosis process during M. tb infection remains to be explored.

## 4. Discussion

M. tb is a successful intracellular pathogen that escapes immune clearance and survives in macrophages. Exploring host responses during M. tb infection by comparative proteomics and transcriptomics are important approaches to understand how M. tb establishes infection. We previously performed proteomic analysis of M. tb-infected macrophages, and some differentially expressed genes (SOD2, IFIT1, and ITIT3) in the proteomics were DE-mRNAs in the Control vs. WT group in this study (Kaewseekhao et al., 2015). It was reported that upregulated RSAD2, IFIT1, and IFIT3 were detected in transcriptomic data of H37Rv-infected mouse macrophages, which was also consistent with our sequencing results (Andreu et al., 2017). Studies have shown that the abundance of ISG15, IFIT1, IFIT2, and IFIT3 proteins increases in microparticles (MPs) released by M. tb-infected human THP-1 cells, which is similar to our results (Hare et al., 2015). Our study found MMP27 is upregulated during H37Rv infection. The matrix metalloproteinase (MMP) family proteins disrupt the extracellular matrix during the disease process, destroying the lung matrix in the lungs, and enhancing the transmission of TB. Previous studies have shown that MMP-1, -3, -7, -9, and -10 are upregulated in the sputum or granulomas from TB patients or macrophages infected with M. tb (Elkington et al., 2011). DNA damage-regulated autophagy modulator 1 (DRAM1) is increased after infection with Mycobacterium marinum (M.m) closely related to M. tb, which can promote the elimination of mycobacteria through selective autophagy (van der Vaart et al., 2014). The lack of DRAM1 accelerates the pyroptotic cell death (Zhang et al., 2020). This evidence indicates that DRAM1 plays a central role in combating mycobacterial infections. DRAM1 is one of the upregulated DE-mRNAs in the Control vs. WT group and may be benefit to macrophage resistance to M. tb. We noticed the downregulated expression of TLR4 in the Control vs. WT group. Previous studies on the expression of TLR4 and its effect on downstream pro-inflammatory cytokines are not completely consistent. Some literatures show that TLR4 is upregulated when stimulated by M. tb or virulence proteins and subsequently activates a series of signaling pathways and increases pro-inflammatory cytokines expression, and some literatures show that TLR4 is downregulated during infection (Saraav et al., 2014; Kim et al., 2018; Shi et al., 2018; Jang et al., 2019; Li and Zhang, 2019). This discrepancy may be caused by the difference in stimulus conditions. BSN is the common target of these three DE-miRNAs (miR-129-1-3p, miR-6772-3p, and miR-135b-3p). The literature shows that miR-708-5p and miR-1178 are upregulated during the process of M. tb infecting macrophages, targeting TLR4 to perform their functions, similar to our sequencing results (Shi et al., 2018; Li and Zhang, 2019). The function of BSN during M. tb infection is unclear. However, previous research on the function of BSN is related to autophagy. It has been shown that knocking out BSN can induce increased levels of autophagy (Okerlund et al., 2017; Montenegro-Venegas et al., 2020, 2021). BSN is an important target gene in the ceRNA networks, which may require more attention. Our findings provide support for further comprehension of the M. tb infection process.

We compared the transcriptome data obtained in this study with previous clinical sample analysis. ENST00000442037, one of the top 25-fold changes DE-lncRNAs in plasma of ATB patients compared with healthy controls, was the DE-lncRNA of the Control vs. WT group in this study (He et al., 2017). The discrepancy between our transcriptome results and clinical samples may be due to differences in sample types, as well as limitations of the THP-1 cell model. THP1-derived-macrophages were similar with hMDM in controlling M. tb growth and pro-inflammatory responses compared with U937 and better obtained than hMDM (Mendoza-Coronel and Castañón-Arreola, 2016). To a certain extent, they are suitable substitutes for hMDM. However, THP-1 cell model is only a simplified macrophage model, and there are still differences in the responses of PMA-differentiated THP-1 macrophages and hMDM after stimulation (Tedesco et al., 2018). In addition, our samples collected 24 h post infection can only reflect the transcriptional profile of the host after short-term stimulation, lacking host non-coding RNA expression data caused by persistent infection.

We found some DE-lncRNAs and DE-circRNAs that can bind multiple DE-miRNAs simultaneously in the network diagrams. In the network diagram during H37Rv infection, there were 24 DE-lncRNAs including 4 newly discovered lncRNAs co-targeting hsa-miR-129-1-3p and hsa-miR-6772-3p. Hsacirc_042476 and hsacirc_004818 co-target hsa-miR-129-1-3p and hsa-miR-135b-3p, and hsacirc_015744 is predicted to bind hsa-miR-129-1-3p and hsa-miR-6772-3p. In the network diagram during H37Rv△1759c infection, there were 17 DE-lncRNAs including 3 novel DE-lncRNAs and 5 DE-circRNAs targeting all upregulated DE-miRNAs. We grouped DE-circRNAs with the same expression trend into the same cluster. Hsacirc_051417, hsacirc_069203, and hsacirc_065830 targeting all upregulated DE-miRNAs during H37Rv△1759c infection are located in one expression cluster cluster9, suggesting that these three DE-circRNAs are regulated by the same pathway. DE-circRNAs in different clusters can also target the same DE-miRNAs, indicating that DE-miRNAs regulated by different signaling pathways can act synergistically. It shows that the regulatory mechanism of ceRNA is complex. What is the mechanism of multiple lncRNAs or circRNAs bind the same miRNA? Whether lncRNAs and circRNAs are required to work together due to the low abundance of lncRNAs and circRNAs is worthy of further exploration. In addition, there are no DE-lncRNA and DE-circRNA in the WT vs. △1759c group predicted to interact with DE-miRNAs and had opposite expression trends. We were unable to construct a regulatory diagram of the ceRNA network closely related to Rv1759c. It is possible that DE-miRNAs closely related to Rv1759c are directly regulated through transcriptional regulators. Our previous finding that Rv1759c can increase M. tb survival by altering miR-25 through NFKBIZ supports the above hypothesis. By analyzing the distribution of DE-noncoding RNAs between the three comparison groups, it was found that hsa-miR-12136, hsa-miR-4485-3p, and hsa-miR-7704 were upregulated in the Control vs. WT groups, downregulated in the WT vs. △1759c group but not changed in the Control vs. △1759c group, suggesting that these three DE-miRNAs are closely and directly related to Rv1759c. In addition, 92 DE-lncRNAs and 26 DE-circRNAs were directly related to Rv1759c (Supplementary Table S9). Deletion of Rv1759c altered a large number of lncRNAs and circRNAs expression, which may function through other mechanisms, such as being translated into polypeptides.

We verified the expression and function of hsa-miR-181b-3p in the ceRNA network after H37Rv infection, and proved that the silencing of hsa-miR-181b-3p can promote the clearance of M. tb in macrophages. We noticed that CXCL8 and CXCL9 are the target genes of hsa-miR-181b-3p. Previous literature showed that the expression of CXCL8 and CXCL9 in the serum of patients with active pulmonary tuberculosis is elevated and returns to normal levels after 4–6 months of treatment (Alessandri et al., 2006). In addition, exogenous CXCL8 can reduce the survival of M. tb in macrophages, and inhibition of CXCL8 is associated with intracellular M. tb proliferation (O'Kane et al., 2007). During host resistance to M. tb infection, upregulated CXCL8 can recruit neutrophils, T lymphocytes, and monocytes, playing an important role in the immune response (Baggiolini, 1998; Silva Miranda et al., 2012). CXCL8 and CXCL9 can not only serve as potential diagnostic markers of TB, but also participate in the process of clearing M. tb. The regulation of hsa-miR-181b-3p on the survival of M. tb in macrophages may be realized through CXCL8 and CXCL9.

PE_PGRS family proteins can affect the expression of cytokines and regulate macrophage function through inflammatory responses. PE_PGRS33, PE_PGRS62, PE_PGRS41, and PE_PGRS18 can decrease pro-inflammatory cytokines expression or increase anti-inflammatory cytokines expression (Xie et al., 2021). In the WT vs. △1759c group, we observed the pro-inflammatory cytokines IL-6, IL-23A, and the IL-1 cytokine family IL36G were downregulated, which could also be observed in heatmap-related immunity. This finding implies that deletion of Rv1759c attenuates the host’s pro-inflammatory response in the course of M. tb infection. In addition, we discovered that the chemokines CXCL1, CXCL2, CXCL11, CXCL13, and CCL26 and the chemokine receptor CCR7 were downregulated in WT vs. △1759c group. During M. tb infection, CXCL1 and CXCL2 showed the most significant accumulation on neutrophils, which are important for cell recruitment and mycobacterial killing (Slight and Khader, 2013). CXCL11 is necessary for the formation of granulomas, and CXCL13 is necessary for the formation of B cell follicles in lungs infected by M. tb and for the correct positioning of T cells in granulomas (Chakravarty et al., 2007; Maglione et al., 2007). CCR7 mediates the effective migration of DCs and the activation of M. tb-specific T cells. These chemokines are indispensable for the formation of granulomas. The gradient of chemokines makes cells migrate along the chemokine gradient from brink to the inside of the granuloma (Domingo-Gonzalez et al., 2016). It has been reported that during M.m infection, the preferentially expressed gene in granulomas is the homologous gene of M. tb PE_PGRS, and deletion of the PE_PGRS homologous gene MMAR_0242 in M.m. reduces bacterial load and prevents the production of granulomas (Ramakrishnan et al., 2000; Singh et al., 2016). This evidence indicates that Rv1759c may affect the formation of M. tb-infected host granulomas by affecting the expression of chemokines and cell migration. This finding also prompted us to investigate the influence of other PE_PGRS family proteins on chemokines.

By analyzing the Venn diagram, we found that 68 DE-mRNAs appeared only due to Rv1759c stimulation, and 22 DE-mRNAs were caused by Rv1759c and other M. tb genes or components. We obtained a total of 90 DE-mRNAs associated with Rv1759c stimulation. Calcium/calmodulin-dependent protein kinase IG (CAMK1G) and CaM kinase-like vesicle associated protein (CAMKV) appear in the DE-mRNAs of the WT vs. △1759c group which was predicted to bind calmodulin and act as calmodulin-dependent protein kinase. It is necessary to increase cytoplasmic calcium in macrophages to promote phagosome maturation and M. tb clearance (Connolly and Kusner, 2007). It was demonstrated that most PE_PGRS family proteins contain the Ca2 + −binding motif and demonstrated that Rv0297 contributes to the release of Ca2+ in cells (Bachhawat and Singh, 2007; Yeruva et al., 2016; Grover et al., 2018). These evidences suggest that the function of Rv1759c may affect the M. tb intracellular survival by binding to calcium or regulating calcium-related proteins. ROS is an important player in controlling M. tb. Patients deficient in NOX2, the NADPH oxidase that produces ROS, known as chronic granulomatous disease, have more difficulty controlling TB (Deffert et al., 2014). SOD2, one of the downregulated DE-mRNAs of the WT vs. △1759c group and related ROS production, a member of the superoxide dismutase family, was demonstrated upregulated during M. tb infection promoting the survival of M. tb in macrophages by reducing ROS production (Ren et al., 2021). This suggests that the deletion of Rv1759c may reduce the M. tb survival ability by down-regulating SOD2 and affecting the production of ROS. The expression of apoptosis-related genes P2RX7 and BIRC3 was significantly downregulated, suggesting that the loss of Rv1759c may reduce the cytotoxicity of M. tb. Our previous study shown that Rv1759c can increase the expression of NFKBIZ, affect the autophagy pathway through NFKBIZ/miR-25/NPC1, and promote the survival of M. tb in macrophages, and NFKBIZ was also associated with ROS and apoptosis signaling pathways. The genes regulated by Rv1759c function through multiple pathways. We found that most of DE-mRNAs did not appear in the ceRNA networks. We think that they may be directly regulated by different activation levels of signaling pathways, which is consistent with our GO and KEGG analyses.

In conclusion, we constructed the ceRNA regulatory networks during H37Rv and H37Rv△1759c infection, analyzed the effect of Rv1759c on host mRNA and non-coding RNA expression, and pointed out the close connection between Rv759c and chemokines. We provide new understanding of the pathogenic mechanism and Rv1759c function during M. tb infection. Based on the data of transcriptome data, we analyzed the lncRNA expression and constructed the ceRNA networks. The subcellular localization of lncRNA has not been carried out, which needed and further analysis and verification. The non-coding RNA regulatory networks constructed in this study based on the negative regulatory model and differentially expressed genes still needs to be verified experimentally. The roles of important differential genes during M. tb infection need to be more fully validated. And the effect of Rv1759c on host immunity needs to be further explored. We are currently conducting follow-up studies on these unresolved mechanisms.

## Data availability statement

The datasets presented in this study can be found in online repositories. The names of the repository/repositories and accession number(s) can be found at: https://www.ncbi.nlm.nih.gov/genbank/, GSE184660.

## Author contributions

TC, HC, and XW designed the experiments. WD, GW, and YB performed the experiments. WD, YL, JZ, HL, WL, and CW analyzed data. XH and WD made heatmaps and network diagrams. TC and WD wrote the manuscript. All authors contributed to the article and approved the submitted version.

## Funding

This work was supported by National Natural Science Foundation of China [32273008], the National Key R&D Program [2021YFD1800402], and the Natural Science Foundation of Hubei Province [2021CFA016].

## Conflict of interest

The authors declare that the research was conducted in the absence of any commercial or financial relationships that could be construed as a potential conflict of interest.

## Publisher’s note

All claims expressed in this article are solely those of the authors and do not necessarily represent those of their affiliated organizations, or those of the publisher, the editors and the reviewers. Any product that may be evaluated in this article, or claim that may be made by its manufacturer, is not guaranteed or endorsed by the publisher.

## Footnotes

^1^ http://ccb.jhu.edu/software/hisat2/index.shtml
