# Supplementary material for: Analysis of the noncoding RNA regulatory networks of H37Rv- and H37Rv△1759c-infected macrophages
Source: Front Microbiol. 2023 Mar 8;14:1106643. doi: 10.3389/fmicb.2023.1106643 (PMC10042141; doi:10.3389/fmicb.2023.1106643)

Figure S1. qRT-PCR detection of the differentially expressed genes post H37Rv or H37Rv△1759c infection. qRT-PCR to verify the expression of CCL20, CXCL20 and IFIT1 (A), qRT-PCR to verify the expression of ENST00000647388, ENST00000445682, and ENST00000666623 (E) , qRT-PCR to verify the expression of hsacirc_036051, hsacirc_041934, and hsacirc_065432 (C), qRT-PCR to verify the expression of hsa-miR-1268a hsa-miR-1271-5p and hsa-miR-4638-5p (G). The relative expression changes of these genes are shown based on the FPKM (fragments per kilobase of exon per million mapped fragments) value in each sample (B, D, F, H).

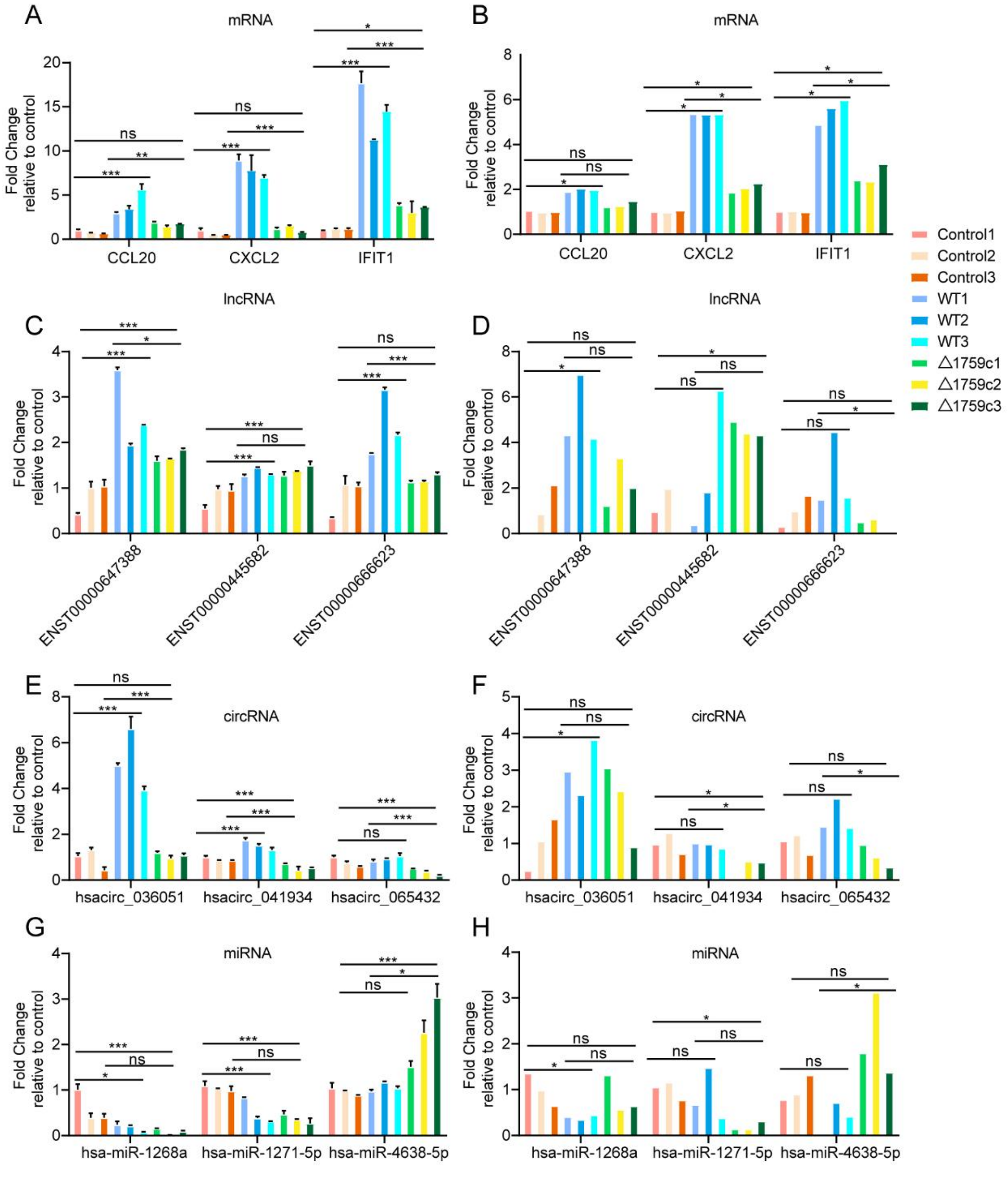

Figure S2. Competing endogenous RNA regulatory networks analysis post H37Rv infection. (A) The lncRNA-miRNA-mRNA regulation network after H37Rv infection. (B) The circRNA-miRNA-mRNA regulation network after H37Rv infection.

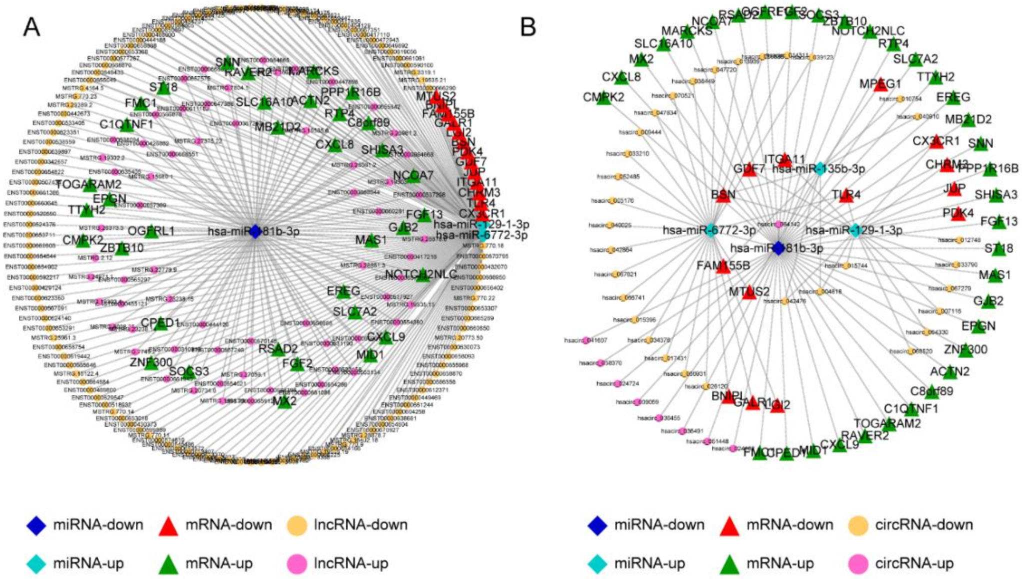

Figure S3. Competing endogenous RNA regulatory networks analysis post H37Rv△1759c infection. (A) The lncRNA-miRNA-mRNA regulation network after H37Rv△1759c infection. (B) The circRNA-miRNA-mRNA regulation network after H37Rv△1759c infection.

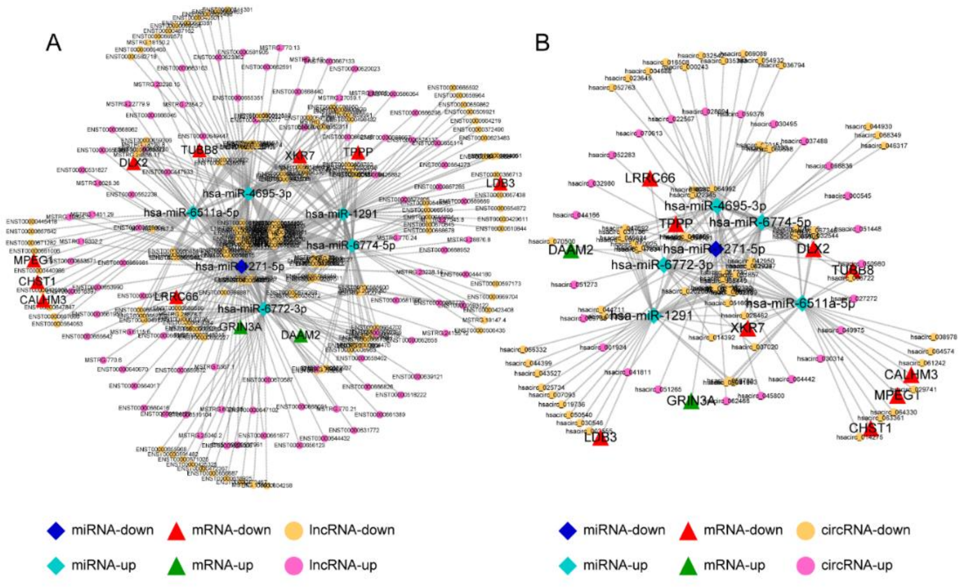

Supplement: Supplementary file 10 [file Data_Sheet_1.PDF]
